# Seizure control during pregnancy and postpartum period in women with epilepsy: an Egyptian prospective study

**DOI:** 10.1186/s12883-023-03086-w

**Published:** 2023-02-02

**Authors:** Hassan Hosny, Manal Mahmoud Al Kattan, Maha A. Zaki, Gihan M. Ramzy, Salsabil Abo Al-Azayem, Rehab Magdy

**Affiliations:** Department of Neurology, Kasr Al-Ainy Faculty of Medicine, Cairo University, Cairo, Egypt

**Keywords:** Women with epilepsy, Seizures, Pregnancy, Postpartum, Anti-seizure medications

## Abstract

**Background:**

Proper seizure control during pregnancy and postpartum is essential to optimize the outcome of women with epilepsy (WWE). The current work aimed to address factors related to seizure occurrence during pregnancy and postpartum.

**Methods:**

One hundred twenty-five WWE, compliant with their anti-seizure medications (ASMs) regimen, were prospectively evaluated for seizure control and ASMs changes all through the pregnancy up to 4 weeks postpartum.

**Results:**

Most of the patients, 73 (58.4%), completed their pregnancy without seizures, while 52 (41.6%) had seizures. Only one case developed one episode of convulsive status epilepticus in the third trimester. Due to breakthrough seizures, the ASM dose was increased from the first to the third trimester in 19.2% of pregnancies, while another ASM was added in 8 pregnancies. Uncontrolled seizures during the six months before pregnancy were associated with a four-fold increase in the risk of seizures during pregnancy (95% CI 2.476–6.695). The latter nearly doubled the risk of seizures during the postpartum period (RR 1.978) (95% CI 1.44 -2.717). Furthermore, genetic etiology would increase the risk of seizures during the postpartum period by 2.7 times more than the unknown etiology (RR 2.778, 95%CI 1.156–6.679).

**Conclusion:**

Women with epilepsy should be counselled that proper seizure control six months before pregnancy is necessary to pass their pregnancy and the postpartum period without seizures.

## Introduction

In the Arab world, the estimated prevalence of epilepsy ranged from 0.9/1000 to 6.5/1000 [1]. Egypt is ranked among the most prevalent Arab countries with epilepsy, where the prevalence of epilepsy was estimated to be 6.9 cases per 1000 [2].

Epilepsy is associated with an overall increased risk of maternal deaths, both during and after pregnancy, and an increased risk of Sudden Unexpected Death in Epilepsy (SUDEP) specifically [3]. Uncontrolled seizures during pregnancy add another risk factor for epilepsy-related deaths [3, 4]. In addition, Y-H Chen et al. [5] found that uncontrolled seizures can partially contribute to adverse pregnancy outcomes in women with epilepsy (WWE), such as preterm delivery and low birth weight. The postpartum period is considered another vulnerable time for seizures, as stress increases and sleep is disturbed [6]. Taken together, every attempt should be made to prevent seizures during pregnancy and postpartum.

That's why several studies have been interested in revealing predictors of seizures during pregnancy [7–9] as well as postpartum seizures in WWE [10, 11].

In this work, Egyptian WWE adhering to the treatment were prospectively followed during the entire pregnancy and up to 4 weeks postpartum to assess seizure control during pregnancy and the postpartum period concerning pre-pregnancy control, age, age at onset, seizure type, etiology, and treatment regimen.

## Methods

The present study was a part of the Egyptian Registry of Anti-seizure medications and Pregnancy system, in which 211 pregnant WWE were prospectively evaluated from November 2018 to November 2020 [12].

Out of 211 pregnancies included in that study, 27 ended prematurely due to intrauterine deaths, five patients started their seizures for the first time during pregnancy, and 54 were noncompliant with their anti-seizure medications (ASMs) regimen. Hence, only 125 WWE were included in the current analysis of seizure control. Only one woman was excluded from the analysis of seizure control during the postpartum period because she delivered a stillbirth.

Data for these patients regarding seizure control and treatment changes during the entire pregnancy and up to four weeks postpartum were prospectively registered into the Egyptian Registry of Anti-seizure medications and Pregnancy system.

Seizure control was verified six months before pregnancy, during each trimester, at the time of delivery, and during the four postpartum weeks. Treatment changes regarding the type or dose of ASMs in each of these previous periods were also documented. Additionally, the occurrence of status epilepticus (SE), either convulsive or non-convulsive, was evaluated.

### Ethical statement

All the participants signed their informed consent to the study. The study was performed following the Helsinki declaration. The ethical committee of the Faculty of Medicine, Cairo University, approved the study (approval number is D-39–2019).

### Statistical analysis

Data were summarized using mean and standard deviation for quantitative data and count and percentages for categorical data. Comparisons between categorical data were made using the Chi-square test. Relative risk (RR) for seizures occurring during pregnancy and postpartum was estimated. *P*-values less than 0.05 were considered statistically significant.

## Results

One hundred twenty-five WWE were prospectively followed during pregnancy. Their ages ranged from 17 to 41 years, with a mean age of 27.3 ± 5.4.

### Overall seizure control and treatment changes during pregnancy

Most of the patients, 73 (58.4%), successfully completed their pregnancy without seizures, while 52 (41.6%) had seizures. Seizure frequency was unchanged in 97 cases (77.6%), of which 73 (75.2%) were seizure-free during the entire pregnancy. Of the remaining 28 pregnancies, six patients (4.8%) had a reduction in seizure frequency in the second and \ or third trimester, and 22 patients (17.6%) had deteriorated.

Only one case developed one episode of convulsive SE in the third trimester. This case was diagnosed as preeclampsia during the third trimester.

Seizure control during pregnancy in relation to different ASMs is illustrated in Table 1. Due to breakthrough seizures, the ASM dose was increased from the first to the third trimester in 19.2% of pregnancies (24/125), while another ASM was added in 8 pregnancies (6.4%).

**Table 1:** Anti-seizure medications used by eligible patients in relation to seizure control during pregnancy

ASMs at conception	Seizures during the entire pregnancy	
	Yes (*n* = 52)	No (*n* = 73)
Valproate monotherapy	2 (3.8%)	4 (5.5%)
Levetiracetam monotherapy	13 (25%)	32 (43.8%)
Carbamazepine monotherapy	9 (17.3%)	14 (19.2%)
Lamotrigine monotherapy	1 (1.9%)	0 (0.0%)
Oxcarbazepine monotherapy	3 (5.8%)	0 (0.0%)
Polytherapy	24 (46.2%)	23 (31.5%)

*ASM****s*** anti-seizure medications

Seizures occurred during delivery in 7 (5.6%) patients, two on monotherapy (one on levetiracetam and another on valproate) and five on polytherapy.

### Risk estimation for seizures occurring during pregnancy

Univariate analysis showed that seizure freedom six months before pregnancy was significantly associated with seizure freedom during pregnancy (*P* < 0.001) (Table 2).

**Table 2:** Comparison between patients who had seizures versus who had no seizures during pregnancy

		Seizures during the entire pregnancy		*P*-value
		Yes (*n* = 52)	No (*n* = 73)	
Age		27.31 ± 5.43	27.23 ± 5.37	0.773
Age at onset		14.16 ± 7.47	14.89 ± 6.73	0.361
Seizure type	Focal	31 (59.6%)	36 (49.3%)	0.255
	Generalized	21 (40.4%)	37 (50.7%)	
Aetiology	Genetic	21 (40.4%)	37 (50.7%)	0.516
	Structural	14 (26.9%)	17 (23.3%)	
	Unknown	17 (32.7%)	19 (26.0%)	
Seizure in 6 months before pregnancy	Controlled	14 (26.9%)	61 (83.6%)	< 0.001*
	Not controlled	38 (73.1%)	12 (16.4%)	
Poly\monotherapy	Monotherapy	28 (53.8%)	50 (68.5%)	0.096
	Polytherapy	24 (46.2%)	23 (31.5%)	

^*^P- value < 0.05 is considered statistically significant

Uncontrolled seizures during the six months before pregnancy were associated with a four-fold increase in the risk of seizures during pregnancy (95% CI 2.476–6.695) (Table 3).

**Table 3:** Risk estimation of seizures occurring during the entire pregnancy

Variables		Seizures during pregnancy		RR	95% CI		*P*-value
		No *n* = 73	Yes *n* = 52		lower	upper	
Seizure in 6 months before pregnancy	Controlled	61 (83.6%)	14 (26.9%)	4.071	2.476	6.695	< 0.001
	Not controlled	12 (16.4%)	38 (73.1%)				

*RR* Relative risk, *CI* confidence interval

*P*- value < 0.05 is considered statistically significant

### Risk estimation for seizures occurring during the postpartum period

By univariate analysis, genetic and structural etiology were significantly associated with seizure occurrence during the postpartum period compared to those with unknown etiology. Moreover, Seizure occurrence during pregnancy was significantly associated with seizure occurrence during the postpartum period (Table 4).

**Table 4:** Comparison between patients who had seizures and who had no seizures during the postpartum period

		Seizures during the postpartum period		*P*-value
		Yes (*n* = 38, %)	No (*n* = 86, %)	
Age		26.47 ± 4.89	27.7 ± 5.54	0.163
Age at onset		14.01 ± 6.19	14.86 ± 7.42	0.635
Seizure type	Focal	16 (42.1%)	51 (59.3%)	0.076
	Generalized	22 (57.9%)	35 (40.7%)	
Etiology	Genetic	22 (57.9%)	35 (40.7%)	0.033*
	Structural	11 (28.9%)	20 (23.3%)	
	Unknown	5 (13.2%)	31 (36.0%)	
Seizures occurrence in pregnancy	None	9 (23.7%)	63 (73.3%)	< 0.001*
	Yes	29 (76.3%)	23 (26.7%)	
Poly\monotherapy	Monotherapy	17 (44.7%)	54 (62.8%)	0.061
	Polytherapy	21 (55.3%)	32 (37.2%)	

^*^P- value < 0.05 is considered statistically significant

Seizure occurrence during pregnancy nearly doubled the risk of seizures during the postpartum period (RR 1.978) (95% CI 1.44 -2.717). Additionally, genetic etiology would increase the risk of seizures during the postpartum period by 2.7 times more than the unknown etiology (RR 2.778, 95%CI 1.156–6.679) (Table 5).

**Table 5:** Risk estimation of seizures occurring during the postpartum period

Variables		Seizures during the postpartum period		RR	95% CI		*P*-value
					Lower	upper	
		No *n* = 86	Yes *n* = 38				
Etiology	Genetic	35 (40.7%)	22 (57.9%)	1.087	0.611	1.936	0.775
	Structural	20 (23.3%)	11 (28.9%)				
	Genetic	35 (40.7%)	22 (57.9%)	2.778	1.156	6.679	0.022
	Unknown	31 (36%)	5 (13.2%)				
	Structural	20 (23.3%)	11 (28.9%)	2.555	0.996	6.552	0.0509
	Unknown	31(36%)	5 (13.2%)				
Seizures during pregnancy	None	63 (73.3%)	9 (23.7%)	1.978	1.440	2.717	< 0.001
	Yes	23 (26.7%)	29 (76.3%)				

*RR* Relative risk, *CI* confidence interval

*P*- value < 0.05 is considered statistically significant

## Discussion

The current study showed that most of the patients (58.4%) successfully passed their pregnancy without breakthrough seizures in a ratio slightly similar to that found in the 2006 EURAP study (58.3%) [13] but less than EURAP 2013 study (66.6%) [14]. The later EURAP analysis excluded women on polytherapy, who are known to be less likely to have complete seizure control [14]. In the Australian register of ASMs in pregnancy, only 50.3% of WWE remained seizure-free throughout the entire pregnancy [8]. Compared with other developing countries, our ratio was higher than the KREP study in which 47.8% of WWE remained seizure-free throughout the entire pregnancy [9]. However, PB Pennell et al. [15] found that the rate of seizure worsening in WWE during and after pregnancy was similar when compared to WWE who were not pregnant and observed over the same time period.

Seizure frequency was unchanged in 77.6% of our study population. This proportion was higher than those reported by EURAP study 2013 [14] and EURAP study 2006 [13] (70.5% and 63.6%), respectively. In addition, the percent of worsening in seizure control in our study population from the first to second or third trimesters was similar to those reported by the EURAP study (17.6% in ours, 17.3% in EURAP 2006 [13] and 15.8% in EURAP 2013 [14].

In this study, SE occurred in only one case (0.8%) with no maternal mortality. According to KR Rajiv and A Radhakrishnan [16], SE occurs in 1–2% of pregnancies in WWE and does not seem to be more frequent than in other periods of life. This finding contrasts with earlier estimates that suggest high maternal mortality rates in SE during pregnancy [17].

In the EURAP study, the drug dose was increased, or a second ASM was added in almost one-third of the pregnancies. These changes in treatment schedule were not limited to patients who had breakthrough seizures but also to patients who were taking ASMs whose levels had decreased during the second and third trimesters of pregnancy to prevent breakthrough seizures [14]. In our study, increases in doses and/or number of ASMs occurred in 25.6% of pregnancies. They were almost in pregnancies with breakthrough seizures, which may be due to the lack of serum level of those AEDs known to be affected during pregnancy (levetiracetam, lamotrigine, oxcarbazepine) [18].

We agreed with all previous studies that pre-pregnancy uncontrolled seizure predicts breakthrough seizure during pregnancy, but the period studied before pregnancy varied from one study to another. While we studied the effect of the six months as a preconception period, J Allotey et al. [7] studied the effect of 3 months, SV Thomas et al. [9] studied the effect of one month before pregnancy, PE Voinescu et al. [10] studied the effect of 9 months and one year was evaluated by FJ Vajda et al. [8].

Therefore, WWE should be counselled to postpone pregnancy until seizures are controlled to avoid breakthrough seizures during pregnancy.

The present study showed that age, age at onset, seizure type, etiology, or treatment regimen were not significantly associated with seizures during pregnancy, in contrast to other registries that found that seizures affected pregnancies occurred in younger mothers, with earlier onset, with focal seizures, and who were exposed to polytherapy [7–10, 14].

In this study, the type of ASM was not studied concerning seizure occurrence during pregnancy due to the limited number of our patients. Larger registries found that the serum level of some ASMs drooped during pregnancy; oxcarbazepine in the EURAP 2006 [13] and lamotrigine in the EURAP 2013 study [14].

The postpartum period is considered physically and emotionally stressful for WWE [6]. Therefore, detecting predictors of seizure occurrence during this vulnerable period is crucial to keep the nursing mother and her baby safe. In agreement with the Australian registry [8], this study showed that seizure occurrence during pregnancy was associated with seizure occurrence during the postpartum period. Therefore, WWE who were controlled during pregnancy should be reassured that they would most likely be safe during the postpartum period.

The present study showed that genetic etiology was associated with seizure occurrence during the postpartum period than those with unknown etiology (*RR* = 2.778). Sleep deprivation, a feature of the postpartum phase, is a known seizure trigger for those with genetic etiology [19]. Therefore, family help and support to assist with night feeding were recommended [6].

Finally, the relatively small size of the study population is the main noteworthy limitation of this study that impeded the analysis of each ASM individually in relation to seizure occurrence during pregnancy. Lack of drug serum level monitoring during pregnancy was another limitation.

## Conclusion

To conclude, most of our patients successfully completed their pregnancy without seizures. Seizure occurrence six months before pregnancy was associated with an increased risk of breakthrough seizure during pregnancy, and the latter was associated with seizure occurrence during the postpartum period.

## Data Availability

Authors report that the datasets used and/or analyzed during the current study are available from the corresponding author on reasonable request.
